# Production Change Optimization Model of Nonlinear Supply Chain System under Emergencies

**DOI:** 10.3390/s23073718

**Published:** 2023-04-04

**Authors:** Jing Zhang, Yingnian Wu, Qingkui Li

**Affiliations:** 1 School of Automation, Beijing Information Science and Technology University (BISTU), Beijing 100192, China; 2 Institute of Intelligent Networked Things and Cooperative Control, Beijing Information Science and Technology University (BISTU), Beijing 100192, China; 3 Intelligent Perception and Control of High-End Equipment Beijing International Science and Technology Cooperation Base, Beijing Information Science and Technology University (BISTU), Beijing 100192, China

**Keywords:** optimization model, adaptive sliding mode control, nonlinear supply chain system, production change, RBF neural network, emergencies

## Abstract

Aiming at the problem that the upstream manufacturer cannot accurately formulate the production plan after the link of the nonlinear supply chain system changes under emergencies, an optimization model of production change in a nonlinear supply chain system under emergencies is designed. Firstly, based on the structural characteristics of the supply chain system and the logical relationship between production, sales, and storage parameters, a three-level single-chain nonlinear supply chain dynamic system model containing producers, sellers, and retailers was established based on the introduction of nonlinear parameters. Secondly, the radial basis function (RBF) neural network and improved fast variable power convergence law were introduced to improve the traditional sliding mode control, and the improved adaptive sliding mode control is proposed so that it can have a good control effect on the unknown nonlinear supply chain system. Finally, based on the numerical assumptions, the constructed optimization model was parameterized and simulated for comparison experiments. The simulation results show that the optimized model can reduce the adjustment time by 37.50% and inventory fluctuation by 42.97%, respectively, compared with the traditional sliding mode control, while helping the supply chain system to return the smooth operation after the change within 5 days.

## 1. Introduction

The supply chain system is a complex network system composed of producers, distributors, and retailers. There is a flow of information such as goods, funds, and orders between each link [1]. It is committed to selling the products through all aspects of the supply chain system. At the same time, when a supply chain system link is compromised, the impact is passed along to the system as a whole, affecting the whole supply chain system [2]. This paper models the supply chain system as a cascading nonlinear system due to the structural characteristics and transferability of the supply chain system. The supply chain system links, such as producers, distributors, and retailers, can be thought of as a specific link of the cascading nonlinear system, and the characteristics of which is used to reflect the relevant characteristics of the supply chain system. When a certain link of the supply chain system breaks under some unexpected emergencies, the supply chain system will not be able to operate normally, and then it is urgent to find a new enterprise to carry out the change, and after the change is completed, the relevant parameters of the supply chain system will change to a certain extent, and then the original production plan is not enough to cope with the existing supply chain system, and then it is necessary to design a new production plan to meet the demand of the supply chain system change. For example, the “Russia-Ukraine conflict” that broke out in 2022: if part of the downstream supply chain system is located in Ukraine, the supply chain system would not be able to function normally, and then the upstream supply chain would need to find new downstream companies to replace them in order to maintain the normal operation of the supply chain system.

The supply chain system is often affected to a certain extent due to emergencies, and failure to respond effectively will cause huge economic losses to the supply chain system. This has led more and more scholars and business managers to conduct research on the optimal management of supply chain systems during emergencies. Xie et al. [3] studied the problem of a significant increase in medical supplies during the new crown epidemic and proposed a multi-party game solution. Wang Limin et al. [4] developed effective pricing strategies for supply chain systems under the effect of uncertain demand disruptions, considering green investments and sales efforts. Chunmei Ma [5] proposed a corresponding multi-objective solution for the closed-loop supply chain system under the dual influence of demand disruption and government subsidies. Fu et al. [6] effectively sought an optimal solution for the problem of input/output constraints and cross-coupling of the interacting nodes of the supply chain system based on distributed model predictive control to help the supply chain system meet the external uncertain demand under unexpected events with the lowest inventory cost. From such references, it can be found that many of them are conducted for satisfying demand perturbations under the impact of unexpected events and do not consider the problem of supply chain system link changes, which is one of the motivations for this paper.

With the trend of globalization and changes in international forms, the traditional way of managing supply chain systems is no longer sufficient to cope with the existing complex market environment. With the rapid development of emerging technologies such as artificial intelligence, digital twin technology, and big data, more and more companies tend to use emerging technologies to optimize the management of supply chain system production [7], transportation [8], product quality [9], material environmental suitability [10], inventory management [11], and customer service [12]. Inspired by the structure of supply chain systems, we model the supply chain system as a cascaded nonlinear system, and for the optimal management of nonlinear systems, sliding mode control has been widely used in the engineering field [13,14,15] to solve nonlinear system control problems because of its simple algorithm and high anti-disturbance capability [16].

Sliding mode control, the dominant method for the controller design of nonlinear systems, was applied to most nonlinear systems [17]. In the primary stage of the development of the sliding mode control, most of the research focused on the design methods of sliding mode control [18,19], and in the middle stage, it mostly focused on the improvement of the sliding mode control convergence laws [20,21,22]. Nowadays, the research related to sliding mode control mainly extends its application area by combining it with other methods, such as adaptive control [23,24,25], neural network algorithms [26,27], and model predictive control [28]. Although sliding mode control has been widely used, there are fewer studies related to optimizing the overall supply chain system management using sliding mode control as known from the literature [29], which is one of the motivations for this paper. Meanwhile, the traditional sliding mode control has the disadvantage of strong jitter [30], and this paper proposed an improved power convergence law based on the traditional power convergence law [31], which can help the supply chain system enterprises converge to the target inventory quickly and reduce the inventory fluctuation after stabilization.

The supply chain system depends on stability. However, if a supply chain system link is affected by unanticipated emergencies, such as the new coronavirus epidemic [32], the Russian–Ukrainian conflict [33], or cyberattacks [34], it will no longer be able to guarantee the supply chain system’s normal operation. In these cases, it will be necessary to find a new company for link changes to guarantee the supply chain system’s normal operation. Unfortunately, relatively little research has been performed in this area, which is another motivation for this paper. The understanding of “engineering change” can be traced back to 1997, when it was first defined by Wringt as the frequent modification of components [35]. The changes indicated in this paper mainly refer to the changes in the supply chain system links, which also caused changes in product design, which is consistent with the definition of engineering changes by Jarratt [36]. It is worth our attention that the current research related to change design is mainly based on static physical models, although some studies are considered to describe the change evolution paths using graph theoretic ideas, for the dynamics on graph theory is hardly involved, which is the last motivation of this paper. A new production plan must be designed to satisfy the market demand once a supply chain system undergoes engineering modifications to the relevant supply chain system characteristics. The more popular RBF neural network is used in this article to approximate the altered unknown linear parameters. Due to their straightforward structure and quick convergence, RBF neural networks were frequently utilized in the study of unknown nonlinear systems [37,38,39,40].

Based on the above research background, this paper mainly studies how to make the manufacturer’s production plan after a change in the supply chain system caused by unexpected events. The contributions of this paper are as follows. Firstly, based on the logical relationship of production–storage–sales and reasonable assumptions, a three-level single-chain nonlinear supply chain system containing producers, distributors, and retailers is established. Secondly, the adaptive improved sliding mode controller is designed using the proposed improved power convergence law and combined with RBF neural network, and then the adaptive improved sliding mode controller is used to construct an optimization model of the supply chain system under unexpected events to help the nonlinear supply chain system to develop a reasonable production plan after the change of links. Finally, the constructed optimization model is used for the simulation experimental analysis to verify the effectiveness of the proposed method in this paper.

The rest of this paper is organized as follows. Section 2 constructs a three-level single-chain nonlinear supply chain system model. We propose a improved adaptive sliding mode control based on an RBF neural network and performs performance analysis in Section 3. Section 4 constructs a three-level single-chain nonlinear supply chain system production change optimization model and conducts stability analysis for the designed controller. We conduct a comparative simulation study based on several examples in Section 5. Section 6 concludes with relevant conclusions and provides an outlook for future research.

## 2. Three-Level Single-Chain Nonlinear Supply Chain System Model

**Figure 1:**
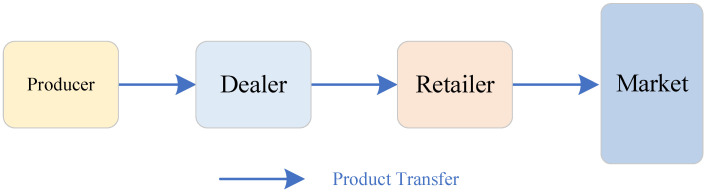
Schematic diagram of three-level single chain tandem supply chain system.

In this paper, based on the current market contains manufacturers, distributors, and retailers in the three aspects of the single chain marketing model establishment and analysis, the structure model as shown in Figure 1, the arrows in the figure represent the product sales route, the product through the supply chain system manufacturers to produce, and then through different links to the corresponding sales area, this paper considers the three-level single chain supply chain system, each link is only from the upstream link to pick up the goods, there is no intermediate link to sell and circulate the goods, and only the retailer is directly facing the market for product sales. At the same time, in most cases, in the actual operation of the supply chain system, the enterprise managers formulate the ’production-storage-sales ’ plan for the day according to the daily demand and inventory.

**Figure 2:**
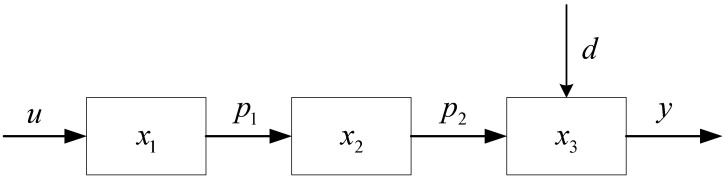
Supply chain system upstream and downstream enterprises inventory production relationship diagram.

Based on the intrinsic linkage of each link of the three-stage single-chain tandem supply chain system shown in Figure 1 and the reference [41], a cyclical multi-level supply chain inventory system dynamic model is established by using the basic quantity logic relationship of “production-storage-sales” for the enterprise inventory management of supply chain system products. Taking the inventory quantity of each link of the supply chain system as the controlled object and considering the parameters such as production quantity of manufacturers, incoming quantity, and outgoing quantity of downstream enterprises, the inventory relationship between upstream and downstream enterprises of the supply chain system can be obtained through equivalence and simplification as shown in Figure 2, where, *x* indicates the inventory level of each link of the supply chain system; *u* represents the production volume of the producer; $p_{1}$ denotes the shipment volume of the producer of the supply chain system to the distributor; $p_{2}$ represents the shipment volume of the distributor of the supply chain system to the retailer; *d* indicates the market demand faced by the retailer of the supply chain system. *y* denotes the quantity of output of the supply chain system.

In this paper, according to the reference [42] the following assumptions are made to be able to model the supply chain inventory system more accurately to more realistically reflect the real situation of the supply chain inventory system.

**Assumption** **1.** *Assume that in the single-chain marketing model, all links only go upstream to pick up goods, and only to a specific downstream for shipment; there is no stringing of goods.*

**Assumption** **2.** *In this paper, we consider the production replenishment time point of the supply chain inventory system is kT, where k=0,1,2,…,n, T represents the production planning cycle. In most cases, the production planning cycle is day, so this paper uses k to represent kT.*

**Assumption** **3.** *Assuming that there is a certain degree of transportation loss in the upstream and downstream transportation process of the supply chain system products, and the transportation loss rate of each link of the supply chain system is δ, then we can obtain:*

(1) $$\begin{array}{c} \delta ={\left[0,\delta_{1},\delta_{2}\right]}^{T} \end{array}$$

**Assumption** **4.** *The product under consideration is perishable, and there is a self-loss of inventory at each stage of the production process due to the product itself or storage conditions, and assume that the self-loss rate of the supply chain inventory system is ρ for a single cycle T, then we can obtain:*

(2) $$\begin{array}{c} \rho ={\left[\rho_{1},\rho_{2},\rho_{3}\right]}^{T} \end{array}$$

**Assumption** **5.** *Assume that shipments outside the retailer in the supply chain system are determined by the inventory levels in the upstream and downstream segments and that the retailer’s shipments are determined by the market.*

**Figure 3:**
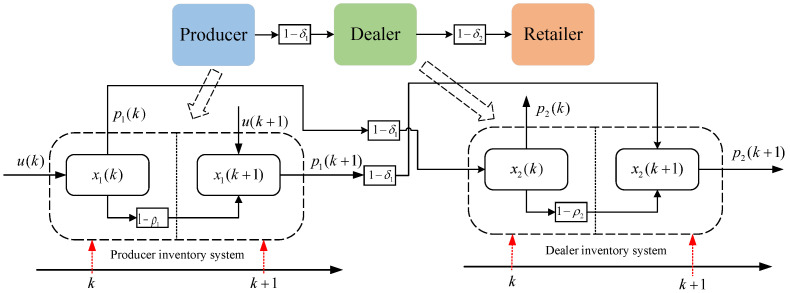
Diagram of the operation of the three-level supply chain inventory system.

Based on the above assumptions, a schematic diagram of the operation of the supply chain inventory system can be obtained as shown in Figure 3.

Figure 3 shows the operation diagram of the three-level supply chain inventory system, which mainly reflects the operation logic of each link of the supply chain system in a cycle *T*. The loss of products in each cycle mainly includes two aspects of self-destruction and transportation loss, and the change of product quantity in each cycle conforms to the basic “production-storage-sales” quantity logic relationship. According to the schematic diagram of the supply chain inventory system in Figure 3 and the basic logical relationship of inventory management in the enterprise of “production-storage-sales” of supply chain system products, the following expressions can be obtained.

(3) $$\begin{array}{c} x_{1}\left(k+1\right)=\left(1-\rho_{1}\right)x_{1}\left(k\right)+u\left(k\right)-p_{1}\left(k\right)\\ x_{2}\left(k+1\right)=\left(1-\rho_{2}\right)x_{2}\left(k\right)+\left(1-\delta_{1}\right)p_{1}\left(k\right)-p_{2}\left(k\right)\\ x_{3}\left(k+1\right)=\left(1-\rho_{3}\right)x_{3}\left(k\right)+\left(1-\delta_{2}\right)p_{2}\left(k\right) \end{array}$$

According to the reference [43], We can transform the inventory function using the Taylor expansion. The Taylor expansion is mainly applicable if the function satisfies certain conditions. The Taylor formula can use the derivative values of the function at a certain point as coefficients to construct a polynomial to approximate the function. Therefore, we can obtain the Taylor expansion of the supply chain system inventory function as follows:

(4) $$\begin{array}{c} x\left(k+h\right)=x\left(t\right)+\frac{x^{\prime }\left(k\right)}{1!}h+\frac{x^{\prime \prime }\left(k\right)}{2!}h^{2}+\cdots +\frac{x^{n}\left(k\right)}{n!}h^{n}+R_{n}\left(k\right). \end{array}$$

where $x^{n}$ is the *n*th-order derivative, n! is the *n*th-order factorial, and *n* is the remainder between the Taylor expansion and the actual value. We consider that when h=1, then can obtain

(5) $$\begin{array}{c} x\left(k+1\right)=x\left(k\right)+\frac{x^{\prime }\left(k\right)}{1!}+R_{1}\left(k\right). \end{array}$$

When $R_{1}\left(t\right)$ is small enough, then it means $R_{1}\left(t\right)\approx 0$, then we can obtain

(6) $$\begin{array}{c} x^{\prime }\left(k\right)=\dot{x}\left(k\right)\approx \frac{x(k+1)-x(t)}{k+1-k}=x\left(k+1\right)-x\left(k\right). \end{array}$$

By Equations (5) and (6) for the inventory of each link of the supply chain system can be expressed as:

(7) $$\begin{array}{c} {\dot{x}}_{1}\left(k\right)=-\rho_{1}x_{1}\left(k\right)+u\left(k\right)-p_{1}\left(k\right)\\ {\dot{x}}_{2}\left(k\right)=-\rho_{2}x_{2}\left(k\right)+\left(1-\delta_{1}\right)p_{1}\left(k\right)-p_{2}\left(k\right)\\ {\dot{x}}_{3}\left(k\right)=-\rho_{3}x_{3}\left(k\right)+\left(1-\delta_{2}\right)p_{2}\left(k\right) \end{array}.$$

According to Assumption A5, the supply chain system distributors’ shipments show a non-linear relationship between their inventory and that of the retailers, and it is also closely related to its own inventory volume. Then we can obtain:

(8) $$p_{2}=f+x_{2}.$$

where *f* is the relevant nonlinear parameter, defined as parameters related to distributor shipments, used to regulate the shipment volume.

In summary, according to Equations (7) and (8) we can obtain the expression of the three-level nonlinear single-chain supply chain system containing producers, distributors, and retailers.

(9) $$\left\{\begin{array}{c} {\dot{x}}_{1}=-\rho_{1}x_{1}+u-p_{1}\\ {\dot{x}}_{2}=-\rho_{2}x_{2}+\left(1-\delta_{1}\right)p_{1}-f-x_{2}\\ {\dot{x}}_{3}=-\rho_{3}x_{3}+\left(1-\delta_{2}\right)f+\left(1-\delta_{2}\right)x_{2}\\ y=x_{3} \end{array}\right.$$

For the constructed three-level nonlinear single-chain supply chain system dynamic model, the following points need to be made in this paper:

**Remark** **1.** *The expression for the nonlinear supply chain inventory system is obtained by introducing nonlinear variables to describe the nonlinear relationships in the supply chain system, based on the traditional inventory parameter logic relationship.*

**Remark** **2.** *The inventory levels of each link in the supply chain system have a range of values and cannot exceed the maximum inventory level of the enterprise.*

**Remark** **3.** *When the inventory level of a link in the supply chain system falls below 0, it indicates that the link is experiencing a stock-out situation, which is a realistic scenario in actual supply chain inventory systems.*

## 3. Improved Adaptive Sliding Mode Control

**Figure 4:**
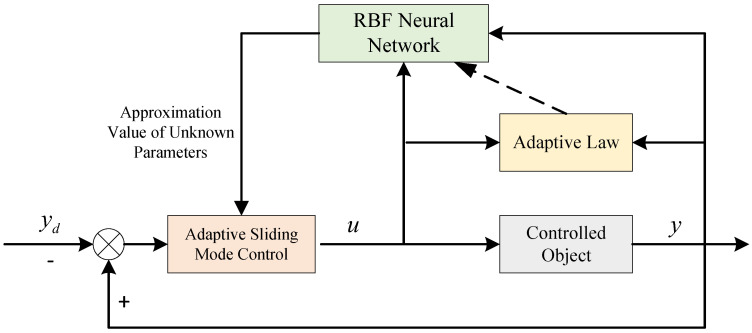
Adaptive sliding mode control based on RBF neural network.

When links in the supply chain system are changed, some of the nonlinear parameters may also change, which poses great challenges to supply chain enterprise managers who cannot confirm the changed parameters. In this study, we propose an adaptive sliding mode control based on the radial basis function (RBF) neural network, which takes advantage of the infinite approximation property of the RBF neural network for nonlinear parameters. By training the RBF neural network with data generated during the operation of the supply chain system, we can approximate the nonlinear parameters after changes in the links. The design block diagram of the proposed method is shown in Figure 4, where $y_{d}$ denotes the market demand, *y* indicates the supply chain system retailer’s shipments, and *u* represents the supply chain system manufacturer’s production volume, which is primarily formulated by the adaptive sliding mode controller.

From Figure 4, it can be found that the adaptive sliding mode control based on RBF neural network mainly consists of two parts: the sliding mode controller and the RBF neural network structure, in which the sliding mode controller mainly designs the corresponding controller based on the control target and structural parameters, while the RBF neural network module mainly uses the parameters obtained from the system operation to approximate some unknown parameters in the system, and then outputs the relevant parameters to the sliding mode controller module for control compensation.

**Figure 5:**
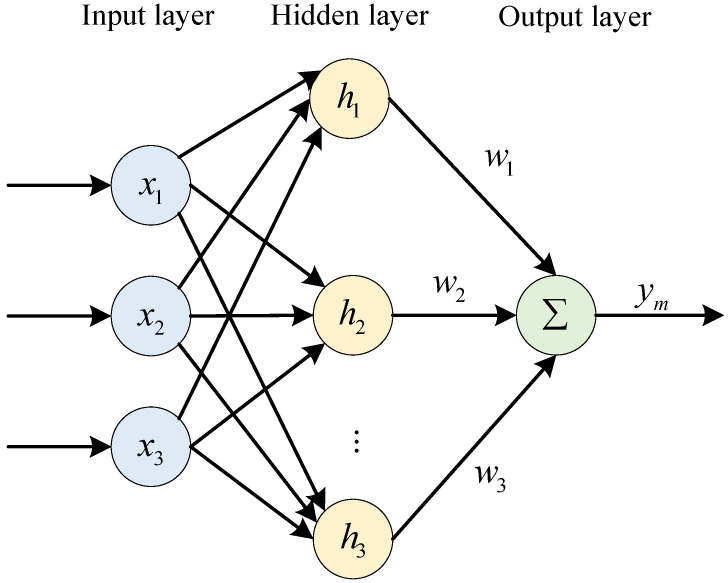
Structure diagram of RBF neural network.

The RBF neural network has good approximation characteristics, and any unknown function can be approximated by enough hidden layer nodes. The structure is shown in Figure 5. The RBF neural network receives the relevant data through the input layer and uses the implicit layer to adjust the parameters and weights to obtain reasonable weight information, which in turn enables the approximation of the nonlinear parameter. For this paper, the main purpose is to use RBF neural network to approximate the adaptive nonlinear function *f*. The inventory *x* in different cycles *T* of the supply chain system is input to the RBF neural network, and the approximation function of the nonlinear function *f* is obtained after training.

In Figure 5, $x={\left[x_{1},x_{2},\cdots x_{n}\right]}^{T}$ is the input vector of the RBF neural network, $H={\left[h_{1},h_{2},\cdots h_{n}\right]}^{T}$ is the radial basis vector of the neural network, and $h_{j}$ is the Gaussian basis function, whose expressions are

(10) $$h_{j}=\exp\left(-\frac{{∥x-c_{j}∥}^{2}}{2b_{j}^{2}}\right),j=1,2,\cdots m.$$

where the center vector of the *j*th node in the hidden layer in the RBF neural network is

(11) $$C_{j}=\left[c_{j1},c_{j1},\cdots c_{jn}\right].$$

The neural network base width vector is

(12) $$B={\left[b_{1},b_{2},\cdots ,b_{m}\right]}^{T}.$$

The neural network output layer weights are

(13) $$W={\left[w_{1},w_{2},\cdots ,w_{m}\right]}^{T}.$$

Then, the complete mapping from the input layer to the output layer can be obtained as

(14) $$y_{m}=\sum_{j=1}^{m}w_{j}H.$$

The mode control algorithm mainly consists of two key elements: the design of the sliding surface and the design of the convergence rate. For the selection of the sliding surface, the design is mainly based on different control situations. The convergence rate design is based on four different traditional convergence rates. In order to be able to improve the problems such as strong jitter and long control time of traditional sliding mode control. In this paper, we propose a fast variable power convergence law, which has faster convergence speed and smaller jitter than the traditional power convergence law. The traditional power convergence law is shown below.

(15) $$\dot{s}=-k\cdot {\left|s\right|}^{\alpha }\cdot \mathrm{sgn}\left(s\right).$$

where 0<k, 0<α<1, and *s* denotes the slip surface function; sgn(s) represents the symbolic function.

To improve the convergence speed of the traditional power convergence law and to reduce the overshoot phenomenon, a improved fast variable power convergence law based on the traditional power convergence law is proposed as follows:

(16) $$\left\{\begin{array}{c} \dot{s}=-k_{1}s-k_{2}q\left(s\right)\mathrm{sgn}\left(s\right)\\ q\left(s\right)=\frac{{\left|s\right|}^{\alpha +1}}{{\left|s\right|}^{\alpha }+a} \end{array}\right..$$

From the improved expression of the fast variable power convergence law, we can see that when the system state is far away from the sliding surface $\left|s\right|\to \infty$, $q\left(s\right)\approx \left|s\right|$, the convergence law is $\dot{s}=-k_{1}s-k_{2}\left|s\right|\mathrm{sgn}\left(s\right)$, which has a faster convergence speed compared with the traditional power convergence law and can greatly reduce the convergence time. When the system is in state $\left|s\right|\to 0$, q(s)≈0, the convergence speed is guaranteed while the system enters the sliding mode section smoothly to eliminate jitter compared to the conventional power convergence law. For the improved fast variable power convergence law, the following analysis is also carried out in this paper.

Firstly, the stability analysis is performed to ensure that the improved fast variable power convergence law can guarantee that the system reaches a stable steady state. From Equation (16), the convergence law is $\dot{s}=-k_{1}s-k_{2}q\left(s\right)\mathrm{sgn}\left(s\right)$, then we can obtain:

(17) $$s\dot{s}=-k_{1}s^{2}-k_{2}q\left(s\right)\leq 0,$$

When and only when s=0, there are $s\dot{s}=0$.

From Equation (17), it is clear that the improved variable power convergence law can help the system state converge to equilibrium point s=0.

Secondly, the jitter property of the improved fast variable power series convergence law after stabilization is analyzed. From Equation (16), when $\left|s\right|\to 0$,$\dot{s}=0$, it shows that the new fast variable power series convergence law can eliminate the jitter of the system after stabilization.

Finally, the convergence rate of the improved fast variable power convergence law is analyzed. Since the traditional power series convergence law only has the power term $-k{\left|s\right|}^{\alpha }\mathrm{sgn}\left(s\right)$ to help the system state converge to the sliding mode steady state, while the improved variable power series convergence law contains $-k_{1}s$ and $-k_{2}q\left(s\right)\mathrm{sgn}\left(s\right)$ to help the system state converge to the sliding mode steady state, which indicates that the improved fast variable power series convergence law with the exponential term has a faster convergence rate.

In summary, through the above three aspects of the analysis of the improved fast variable power convergence law, it can be found that the improved fast variable power convergence law has a better control effect than the traditional power convergence law, both in terms of convergence rate and the system jitter after stabilization.

The improved adaptive sliding mode control based on RBF neural network proposed in this section can not only approximate the nonlinear parameters of the changed supply chain system using RBF neural network but also help the supply chain system converge to the target inventory quantity quickly and weaken the fluctuation problem of inventory after stabilization using the improved fast variable power convergence law.

## 4. Methods and Analysis

For the supply chain system, the upstream and downstream companies must be able to operate stably. However, in the modern supply chain system, the supply chain system may be blocked or even broken due to unexpected events such as the Corona Virus Disease 2019 (COVID-19), the Russia–Ukraine conflict, and natural disasters. In this case, it is necessary to find new downstream companies to change the supply chain system to maintain the stability of the supply chain system and maintain the normal sales of products. The change of the downstream enterprises of the supply chain system will inevitably cause a change in the relevant parameters of the supply chain system, and the production plan of the original upstream manufacturers of the supply chain may not be sufficient to ensure the normal operation of the supply chain system, which will lead to the supply chain system unable to effectively respond to the market demand and cause the problems of shortage of goods or backlog of goods in the upstream and downstream enterprises of the supply chain. This paper designs a production change optimization model for the changed supply chain system, which can help the supply chain system to return to normal operation quickly by making a reasonable production plan according to the changed supply chain system, to meet the downstream and market demand of the supply chain system, and at the same time, it can effectively reduce the inventory fluctuation of the enterprise after the system is stabilized and reduce the waste of enterprise resources.

In order to more rigorous research and analysis, the following assumptions and remarks are required:

**Assumption** **6.** *Assume that the unexpected event does not cause a change in the inventory self-loss rate parameter ρ and transportation loss parameter γ of the supply chain system.*

**Remark** **4.** *In the actual supply chain, due to unexpected emergencies and change propagation effects, there may be multiple downstream changes in the supply chain system. However, in this paper, the research object is a three-level supply chain system, and the change of the middle link distributor will cause the change of both upstream and downstream structures, which is representative. To facilitate the study, we only consider the case of one change in the downstream distributors of the three-level nonlinear single-chain supply chain system in this paper.*

### 4.1. Production Change Optimization Model

From the supply chain system structure considered in this paper, it can be seen that the distributor link is located in the middle of the supply chain system. When the distributor link changes, it will affect the upstream and downstream systems of the supply chain, and at this time, the original production plan of the supply chain manufacturer *u* is not enough to meet the changed supply chain system, which leads to the failure of the supply chain system to operate normally. At this time, the enterprise manager needs to make a new production plan $\hat{u}$ according to the supply chain system after the change of distributors in order to meet the market demand of the supply chain system and ensure the positive operation of the supply chain system.

**Figure 6:**
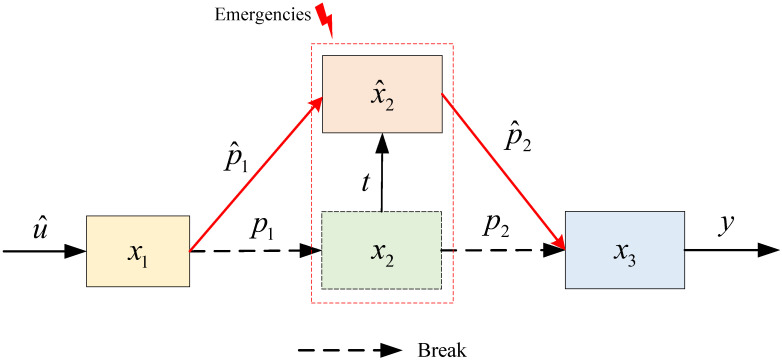
Diagram of the change of distributors in the three-level supply chain system.

Based on the above analysis and the structure of the supply chain system represented in Figure 6, we can obtain the schematic diagram of the change of parameters and structure of the supply chain at time *t* after the change of distributors, as shown in Figure 6.

It can be seen from Figure 6 that at the moment *t*, due to an unexpected event, the distributors of the supply chain system are changed, which leads to some changes in the inventory and structural parameters of the distributors after the change: $x_{2}$ inventory changes from ${\hat{x}}_{2}$; the part connected to the producer changes from $p_{1}$ to ${\hat{p}}_{1}$; and the non-linear parameters connected to the retailer also change, so that $p_{2}$ becomes ${\hat{p}}_{2}$, while the inventory of the other parts of the supply chain system. There is no abrupt change in the inventory level of the other links of the supply chain system. In this paper, let the changed production plan be $\hat{u}$, and from Equation (8), we can obtain

(18) $${\hat{p}}_{2}=\hat{f}+{\hat{x}}_{2}.$$

In summary, we can obtain the changed three-level nonlinear single-chain supply chain system by combining Equations (9) and (18) as

(19) $$\left\{\begin{array}{c} {\dot{x}}_{1}=-\rho_{1}x_{1}+\hat{u}-{\hat{p}}_{1}\\ {\dot{\hat{x}}}_{2}=-\rho_{2}{\hat{x}}_{2}+\left(1-\delta_{1}\right){\hat{p}}_{1}-\hat{f}+{\hat{x}}_{2}\\ {\dot{x}}_{3}=-\rho_{3}x_{3}+\left(1-\delta_{2}\right)\hat{f}+\left(1-\delta_{2}\right){\hat{x}}_{2}\\ y=x_{3}\;. \end{array}\right.$$

**Figure 7:**
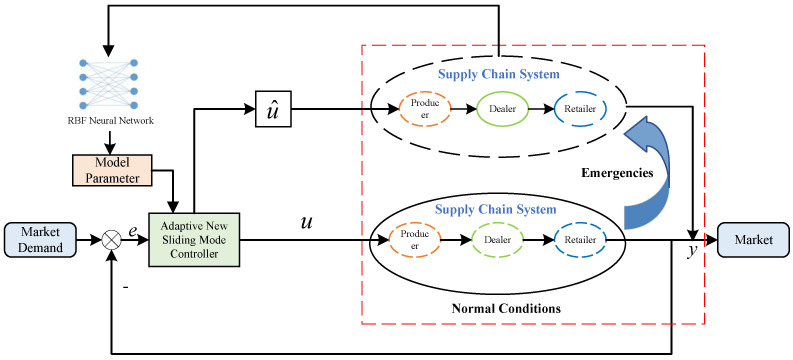
Schematic diagram of production change optimization model for nonlinear supply chain system.

In order to cope with the change of supply chain distributors under unexpected emergencies and help supply chain system producers to formulate effective production strategies, this paper proposes a three-level nonlinear single-chain supply chain system production change optimization model, and the principle diagram of the optimization model is shown in Figure 7.

From Figure 7, it can be seen that the solution of switching production after the change of supply chain system mainly consists of two parts: adaptive improved sliding mode controller and RBF neural network. The improved adaptive sliding mode controller can quickly develop the production plan of the changed supply chain manufacturer based on the model parameters obtained from the RBF neural network, to solve the production problem of the changed supply chain system under the complex environment and reduce the fluctuation of the inventory in the stabilized supply chain system at the same time.

In Figure 7, *y* represents the output of the supply chain system; *u* indicates the producer production under normal conditions; *e* stands for the inventory error of the supply chain inventory system; $\hat{u}$ represents the producer production after the change of distributors. The operational logic of the production change optimization model for a three-level nonlinear supply chain system is as follows. We obtain the market demand *d* faced by the supply chain system in real-time and make a difference with the output *y* of the retailer link of the supply chain system to obtain the error *e*. When the error e=0, it shows that the supply chain single-chain three-level nonlinear supply chain system can meet the market demand. At the same time, the sliding mode controller *u* is designed by using the error *e*, and then the production quantity *u* of the manufacturer of the supply chain system is output. After the change of the dealer in the supply chain system, the RBF neural network is used to obtain the nonlinear function $\hat{f}$ of the changed supply chain system, which is input into the improved sliding mode controller to obtain the manufacturer’s production $\hat{u}$ after the change of the dealer in the supply chain system.

### 4.2. Controller Design

This section focuses on the design of a controller based on adaptive improved sliding mode control with RBF neural network and stability analysis by constructing Lyapunov functions. The controller design is divided into two parts: the design of controller *u* under normal conditions and the design of controller $\hat{u}$ for production changes under unexpected emergencies.

The first is the design of controller *u* for the normal operation of the supply chain system. Through market research, it can be found that many producers in the supply chain system will adopt the “Make-to-Order (MTO)” strategy, that is, the enterprises will make production arrangements according to the demand and delivery date of customer orders andensure that the producer or ’s own inventory is always zero, thereby reducing inventory costs. In this paper, the following assumptions are made for the producers in the three-tier supply chain system.

**Assumption** **7.** *Assume that the manufacturer in the supply chain system uses the “MTO strategy”, in which production is based on downstream orders, and the inventory $x_{1}$ is always equal to zero.*

Then we can obtain the expression of the three-level nonlinear supply chain inventory system after adopting the “MTO strategy”.

(20) $$\;\left\{\begin{array}{c} u=p_{1}\\ {\dot{x}}_{2}=-\rho_{2}x_{2}+\left(1-\delta_{1}\right)p_{1}-f-x_{2}\\ {\dot{x}}_{3}=-\rho_{3}x_{3}+\left(1-\delta_{2}\right)f+\left(1-\delta_{2}\right)x_{2}\\ y=x_{3} \end{array}\right.$$

Then

(21) $$\left\{\begin{array}{c} {\dot{x}}_{2}=-\rho_{2}x_{2}+\left(1-\delta_{1}\right)u-f-x_{2}\\ {\dot{x}}_{3}=-\rho_{3}x_{3}+\left(1-\delta_{2}\right)f+\left(1-\delta_{2}\right)x_{2}\\ y=x_{3} \end{array}\right.$$

According to the inherent characteristics of the supply chain system, the output of the retailer must be able to meet the market demand in order to ensure the stable operation of the supply chain system, this paper will set the market demand to *d*. For the market demand, we also need to make the following explanation:

**Remark** **5.** *In this paper, we only consider the case of fixed market demand, i.e., the market demand is relatively fixed in different sales regions. At the same time, unexpected emergencies do not affect the change of market demand. That is, the fixed market demand is the same before and after the occurrence.*

Define the sales error as the difference between the market demand and the retailer’s output, set to *e*, then we have

(22) $$e=d-y=d-x_{3}\;.$$

We define the slipform surface as

(23) $$s=ce+\dot{e}\;.$$

where *c* is a known constant.

According to Equation (23), we can obtain

(24) $$s=ce+\dot{e}\;.$$

Then, combining Equations (21) and (24), we can obtain

(25) $$\dot{e}=\dot{d}-\dot{y}=\dot{d}-{\dot{x}}_{3}=\dot{d}+\rho_{3}x_{3}-\left(1-\delta_{2}\right)\left(f+x_{2}\right)\;.$$

By using Equation (24), we can have

(26) $$\begin{array}{c} \ddot{e}=\ddot{d}+\rho_{3}{\dot{x}}_{3}-\left(1-\delta_{2}\right)\left(f^{\prime }+{\dot{x}}_{2}\right)=\ddot{d}+\rho_{3}\left[-\rho_{3}x_{3}+\left(1-\delta_{2}\right)\left(f+x_{2}\right)\right]\\ -\left(1-\delta_{2}\right)f^{\prime }-\left(1-\delta_{2}\right)\left[-\rho_{2}x_{2}+\left(1-\delta_{1}\right)u-f-x_{2}\right]\\ =\ddot{d}+\left(1-\delta_{2}\right)\rho_{3}\left(f+x_{2}\right)-{\rho_{3}}^{2}x_{3}-\left(1-\delta_{2}\right)f^{\prime }\\ +\left(1-\delta_{2}\right)\left(\rho_{2}+1\right)x_{2}-\left(1-\delta_{2}\right)\left(1-\delta_{1}\right)u+\left(1-\delta_{2}\right)f\\ =\left(1-\delta_{2}\right)\left(\rho_{3}+\rho_{2}+1\right)x_{2}+\left(1+\rho_{3}\right)\left(1-\delta_{2}\right)f\\ -\left(1-\delta_{2}\right)f^{\prime }+\ddot{d}-{\rho_{3}}^{2}x_{3}-\left(1-\delta_{2}\right)\left(1-\delta_{1}\right)u\;. \end{array}$$

Based on Equations (23), (25) and (26), we know

(27) $$\begin{array}{c} \dot{s}=c\dot{e}+\ddot{e}\\ =c[\dot{d}+\rho_{3}x_{3}-\left(1-\delta_{2}\right)\left(f+x_{2}\right)]\\ +\left(1-\delta_{2}\right)\left(\rho_{3}+\rho_{2}+1\right)x_{2}+\left(1+\rho_{3}\right)\left(1-\delta_{2}\right)f\\ -\left(1-\delta_{2}\right){\hat{f}}^{\prime }+\ddot{d}-{\rho_{3}}^{2}x_{3}-\left(1-\delta_{1}\right)\left(1-\delta_{2}\right)u\;. \end{array}$$

Finally, this paper uses the improved exponential convergence law for the setting of the sliding mode controller, which is obtained from Equations (16) and (27).

(28) $$\begin{array}{c} \dot{s}=c\left[\dot{d}+\rho_{3}x_{3}-\left(1-\delta_{2}\right)\left(f+x_{2}\right)\right]+\left(1-\delta_{2}\right)\left(\rho_{3}+\rho_{2}+1\right)x_{2}+\left(1+\rho_{3}\right)\left(1-\delta_{2}\right)f\\ -\left(1-\delta_{2}\right)f^{\prime }+\ddot{d}-{\rho_{3}}^{2}x_{3}-\left(1-\delta_{1}\right)\left(1-\delta_{2}\right)u=-k_{1}s-k_{2}q\left(s\right)\mathrm{sgn}\left(s\right) \end{array}$$

Solving Equation (28), we can obtain controller *u* for the normal operation of the three-level single-chain nonlinear supply chain system as

(29) $$\left\{\begin{array}{c} u={\left[\left(1-\delta_{1}\right)\left(1-\delta_{2}\right)\right]}^{-1}\{c\left[\dot{d}+\rho_{3}x_{3}-\left(1-\delta_{2}\right)\left(f+x_{2}\right)\right]\\ +\left(1-\delta_{2}\right)\left(\rho_{3}+\rho_{2}+1\right)x_{2}+\left(1+\rho_{3}\right)\left(1-\delta_{2}\right)f\\ -\left(1-\delta_{2}\right)f^{\prime }+\ddot{d}-{\rho_{3}}^{2}x_{3}+k_{1}s+k_{2}q\left(s\right)\mathrm{sgn}\left(s\right)\}\\ q\left(s\right)=\frac{{\left|s\right|}^{\alpha +1}}{{\left|s\right|}^{\alpha }+a}\;. \end{array}\right.$$

Second, when an unexpected event occurs, the design of the production change controller $\hat{u}$ is changed under the unexpected event.

From Equation (19), we can know the dynamic equation of the three-stage single-chain nonlinear supply chain system after the change of distributors, and by associating Equations (19) and (27), we can similarly solve the production plan of the supply chain manufacturer after the change as

(30) $$\left\{\begin{array}{c} \hat{u}={\left[\left(1-\delta_{1}\right)\left(1-\delta_{2}\right)\right]}^{-1}\{c\left[\dot{d}+\rho_{3}x_{3}-\left(1-\delta_{2}\right)\left(\hat{f}+{\hat{x}}_{2}\right)\right]\\ +\left(1-\delta_{2}\right)\left(\rho_{3}+\rho_{2}+1\right){\hat{x}}_{2}+\left(1+\rho_{3}\right)\left(1-\delta_{2}\right)\hat{f}\\ -\left(1-\delta_{2}\right){\hat{f}}^{\prime }+\ddot{d}-{\rho_{3}}^{2}x_{3}+k_{1}s+k_{2}q\left(s\right)\mathrm{sgn}\left(s\right)\}\\ q\left(s\right)=\frac{{\left|s\right|}^{\alpha +1}}{{\left|s\right|}^{\alpha }+a}\;. \end{array}\right.$$

Since the changed nonlinear parameter $\hat{f}$ is unknown, this paper uses RBF neural network for infinite approximation. Let the approximation function $\hat{f}$ be $\hat{F}$, which can be obtained from Equation (14).

(31) $$\hat{F}=\sum_{j=1}^{m}{\hat{w}}_{j}H_{f}={\hat{W}}^{T}H_{f}$$

where, *W* is the ideal RBF neural network weight when approximating the nonlinear parameters, and $H_{f}$ is the Gaussian basis function of the RBF neural network. Then, the production scheme $\hat{u}$ for introducing the approximation function of the RBF neural network can be obtained as

(32) $$\left\{\begin{array}{c} \hat{u}={\left[\left(1-\delta_{1}\right)\left(1-\delta_{2}\right)\right]}^{-1}\{c\left[\dot{d}+\rho_{3}x_{3}-\left(1-\delta_{2}\right)\left(\hat{F}+{\hat{x}}_{2}\right)\right]\\ +\left(1-\delta_{2}\right)\left(\rho_{3}+\rho_{2}+1\right){\hat{x}}_{2}+\left(1+\rho_{3}\right)\left(1-\delta_{2}\right)\hat{F}\\ -\left(1-\delta_{2}\right){\hat{F}}^{\prime }+\ddot{d}-{\rho_{3}}^{2}x_{3}+k_{1}s+k_{2}q\left(s\right)\mathrm{sgn}\left(s\right)\}\\ q\left(s\right)=\frac{{\left|s\right|}^{\alpha +1}}{{\left|s\right|}^{\alpha }+a}\\ \hat{F}=W^{T}H_{f}\;. \end{array}\right.$$

Thus, the design of a improved adaptive sliding mode controller based on the RBF neural network is completed.

### 4.3. Stability Analysis

For the improved adaptive sliding mode controller based on RBF neural network designed above, the stability analysis of the controller is carried out by constructing a Lyapunov function.

First, for the stability analysis of controller *u* under normal conditions, the stability analysis is performed by constructing Lyapunov functions *V*.

(33) $$V=\frac{1}{2}s^{2}$$

Then you can obtain

(34) $$\left\{\begin{array}{c} \dot{V}=s\dot{s}=-k_{1}s^{2}-k_{2}q\left(s\right)\leq 0\\ q\left(s\right)=\frac{{\left|s\right|}^{\alpha +1}}{{\left|s\right|}^{\alpha }+a}\;. \end{array}\right.$$

That is the designed controller is stable under normal conditions.

Second, Controller 1 designed after the change is proved to be stable. In this paper, we take the case where the dealership is changed as an example, and due to the introduction of RBF neural network to approximate the changed nonlinear parameters, it is known

(35) $$\hat{f}=\sum_{j=1}^{m}w_{j}H_{f}+\varepsilon_{f}=W^{T}H_{f}+\varepsilon_{f}\;.$$

Then we can obtain

(36) $$\begin{array}{c} \Delta f=\hat{F}-\hat{f}={\hat{W}}^{T}H_{f}-W^{T}H_{f}+\varepsilon_{f}={\tilde{W}}^{T}H_{f}+\varepsilon_{f}\;, \end{array}$$

where $\tilde{W}=\hat{W}-W$.

When the supply chain system distributor is changed, according to Equations (27) and (32) we can get

(37) $$\begin{array}{c} \dot{s}=c\dot{e}+\ddot{e}=c\left[\dot{d}+\rho_{3}x_{3}-\left(1-\delta_{2}\right)\left(\hat{f}+{\hat{x}}_{2}\right)\right]\\ +\left(1-\delta_{2}\right)\left(\rho_{3}+\rho_{2}+1\right){\hat{x}}_{2}+\left(1+\rho_{3}\right)\left(1-\delta_{2}\right)\hat{f}-\left(1-\delta_{2}\right){\hat{f}}^{\prime }\\ +\ddot{d}-{\rho_{3}}^{2}x_{3}-\left(1-\delta_{1}\right)\left(1-\delta_{2}\right)\hat{u}\\ =c\left[\dot{d}+\rho_{3}x_{3}-\left(1-\delta_{2}\right)\left(\hat{f}+{\hat{x}}_{2}\right)\right]+\left(1-\delta_{2}\right)\left(\rho_{3}+\rho_{2}+1\right){\hat{x}}_{2}+\left(1+\rho_{3}\right)\left(1-\delta_{2}\right)\hat{f}\\ -\left(1-\delta_{2}\right){\hat{f}}^{\prime }+\ddot{d}-{\rho_{3}}^{2}x_{3}-\{c\left[\dot{d}+\rho_{3}x_{3}-\left(1-\delta_{2}\right)\left(\hat{F}+{\hat{x}}_{2}\right)\right]\\ +\left(1-\delta_{2}\right)\left(\rho_{3}+\rho_{2}+1\right){\hat{x}}_{2}+\left(1+\rho_{3}\right)\left(1-\delta_{2}\right)\hat{F}\\ -\left(1-\delta_{2}\right){\hat{F}}^{\prime }+\ddot{d}-{\rho_{3}}^{2}x_{3}+k_{1}s+k_{2}q\left(s\right)\mathrm{sgn}\left(s\right)\}\\ =c\left(1-\delta_{2}\right)\left(\hat{F}-\hat{f}\right)-\left(1+\rho_{3}\right)\left(1-\delta_{2}\right)\left(\hat{F}-\hat{f}\right)\\ +\left(1-\delta_{2}\right)\left({\hat{F}}^{\prime }-{\hat{f}}^{\prime }\right)-k_{1}s-k_{2}q\left(s\right)\mathrm{sgn}\left(s\right) \end{array}$$

This paper constructs the Lyapunov function *L* for the changed function as follows

(38) $$L=\frac{1}{2}s^{2}+\frac{1}{2}\cdot \lambda {\tilde{W}}^{T}\tilde{W}\;.$$

where λ>0 , then we can obtain

(39) $$\begin{array}{c} \dot{L}=s\dot{s}+\lambda {\tilde{W}}^{T}\dot{\tilde{W}}\\ =s\dot{s}+\lambda {\tilde{W}}^{T}\dot{\tilde{W}}=s\dot{s}+\lambda {\tilde{W}}^{T}{\dot{\hat{W}}}^{T}-\lambda {\tilde{W}}^{T}{\dot{W}}^{T}\\ =s\{c\left(1-\delta_{2}\right)\left(\hat{F}-\hat{f}\right)-\left(1+\rho_{3}\right)\left(1-\delta_{2}\right)\left(\hat{F}-\hat{f}\right)\\ -\left(1-\delta_{2}\right)\left({\hat{F}}^{\prime }-{\hat{f}}^{\prime }\right)-k_{1}s-k_{2}q\left(s\right)\mathrm{sgn}\left(s\right)\}-\lambda {\tilde{W}}^{T}{\dot{\hat{W}}}^{T}\\ =s\left(c-1-\rho_{3}\right)\left(1-\delta_{2}\right)\left(\hat{F}-\hat{f}\right)-k_{1}s^{2}-k_{2}q\left(s\right)\\ +s\left(1-\delta_{2}\right)\left({\hat{F}}^{\prime }-{\hat{f}}^{\prime }\right)-\lambda {\tilde{W}}^{T}{\dot{\hat{W}}}^{T}\\ =s\left(c-1-\rho_{3}\right)\left(1-\delta_{2}\right)\left({\tilde{W}}^{T}H_{f}+\varepsilon_{f}\right)\\ -k_{1}s^{2}-k_{2}q\left(s\right)-s\left(1-\delta_{2}\right)\left({\dot{\hat{W}}}^{T}H_{f}\right)-\lambda {\tilde{W}}^{T}{\dot{\hat{W}}}^{T}\\ =\left[s\left(c-1-\rho_{3}\right)\left(1-\delta_{2}\right)H_{f}-\lambda {\dot{\hat{W}}}^{T}\right]{\tilde{W}}^{T}\\ +s\left(c-1-\rho_{3}\right)\left(1-\delta_{2}\right)\varepsilon_{f}\\ -k_{1}s^{2}-k_{2}q\left(s\right)-s\left(1-\delta_{2}\right)\left({\dot{\hat{W}}}^{T}H_{f}\right)\;. \end{array}$$

By solving Equation (39), we can obtain that, if we let the adaptive law be:

(40) $${\dot{\hat{W}}}^{T}=\frac{s\left(c-1-\rho_{3}\right)\left(1-\delta_{2}\right)H_{f}}{\lambda }\;.$$

Then

(41) $$\begin{array}{c} \dot{L}=s\dot{s}+\lambda {\tilde{W}}^{T}\dot{\tilde{W}}\\ =+s\left(c-1-\rho_{3}\right)\left(1-\delta_{2}\right)\varepsilon_{f}-k_{1}s^{2}-k_{2}q\left(s\right)-s\left(1-\delta_{2}\right)\left(\frac{s\left(c-1-\rho_{3}\right)\left(1-\delta_{2}\right)H_{f}}{\lambda }H_{f}\right)\\ =-k_{1}s^{2}-k_{2}q\left(s\right)+\frac{\frac{\lambda \varepsilon_{f}}{\left|s\right|}-\left(c-1-\rho_{3}\right)\left(1-\delta_{2}\right)H_{f}^{2}}{\lambda }s^{2}\left(c-1-\rho_{3}\right)\left(1-\delta_{2}\right)\;. \end{array}$$

Since $-k_{1}s^{2}-k_{2}q\left(s\right)\leq 0$ always holds, it follows that to make $\dot{L}<0$ , it is sufficient to satisfy

(42) $$\left\{\begin{array}{c} \left(c-1-\rho_{3}\right)\left(1-\delta_{2}\right)>0\\ \frac{\lambda \varepsilon_{f}}{\left|s\right|}-\left(c-1-\rho_{3}\right)\left(1-\delta_{2}\right)H_{f}^{2}<0 \end{array}\right.\;.$$

Solving Equation (42), we can obtain

(43) $$\left\{\begin{array}{c} c>1+\rho_{3}\\ c>\frac{\lambda \varepsilon_{f}}{\left|s\right|\left(1-\delta_{2}\right)H_{f}^{2}}+1+\rho_{3} \end{array}\right.\;.$$

From Equation (43), we can obtain that when $\varepsilon_{f}$ is small enough, as long as $c>1+\rho_{3}$ is satisfied, then there will be $\dot{L}<0$, when the design of the changed supply chain producer production controller $\hat{u}$ is stable.

In summary, we can obtain that the controller is stable after the change.

## 5. Simulation and Comparative Analysis

Due to the limitation of experimental conditions and the starting point of this paper is to provide theoretical solutions and ideas for the change management of such supply chain systems, this paper only conducts numerical simulation experiments to show that the optimization model designed in this paper can solve such problems in nonlinear supply chain systems. Based on the MATLAB/Simulink platform, this paper establishes a production change optimization model of supply chain system under emergencies and conducts simulation comparison experiments. The specific experimental steps include: Firstly, the parameters of self-damage rate, transportation loss rate, and shipment regulation are determined based on the assumed supply chain system values to build a three-level single-chain nonlinear supply chain system model including producers, distributors, and retailers, and to build a production change optimization model of the supply chain system under unexpected emergencies, mainly including RBF neural network links, improved sliding mode controller links and three-level nonlinear supply chain system links. Second, the production change optimization model of the supply chain system under unexpected emergencies is parameterized and the optimal combination of control parameters matching the dynamic inventory system of the supply chain is selected to optimize the control effect of the model and improve the control accuracy. Finally, simulation experiments were conducted based on the constructed three-level single-chain nonlinear supply chain system model as well as the optimization model, and the simulation results are comprehensively compared and analyzed.

### 5.1. Parameter Tuning of the Optimization Model

For the optimization model of production changes in supply chain systems under contingencies, parameter rectification is also crucial. The parameter adjustment for the model is mainly to adjust the parameters of the designed controller. The parameters of good control can effectively shorten the control time and improve the system response speed to achieve a better control effect. In this paper, the SIMULINK platform was used for the experiments of model parameters rectification, and the fixed market demand is adopted as the target output quantity of the supply chain system. In this paper, we assume that the parameters of a three-level single-chain nonlinear supply chain inventory system model are shown as follows.

$$\rho ={\left[0.014\;;\;0.022\;;\;0.028\right]}^{T}\;\;\;\;\;\delta ={\left[0\;;\;0.002\;;\;0.004\right]}^{T}$$

$$f=x_{1}^{2}+{\left(x_{2}\right)}^{\frac{1}{2}}-{\left(x_{3}\right)}^{\frac{3}{2}}\;\;\;\;\;d=1.5$$

In this paper, the parameters of the optimization model are rectified and optimized using the supply chain system under normal conditions, in which the initial parameters of the controller for the optimization model of the production change of the supply chain system under unexpected emergencies are set as shown in Table 1.

**Figure 8:**
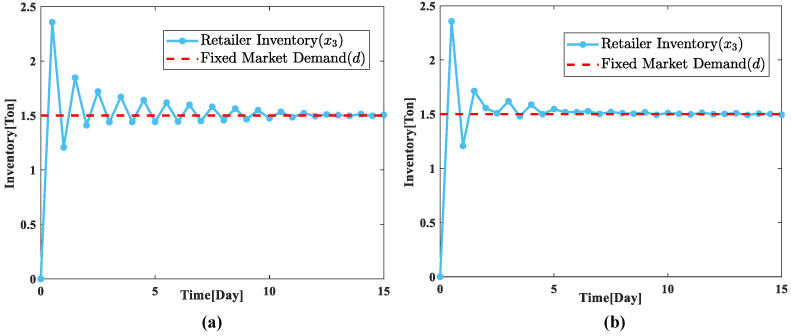
Diagram of the change in retailer’s inventory before and after parameter adjustment. (**a**) Initial parameters; (**b**) optimization parameters.

We take the retailers of the supply chain system as the reference object and conduct the parameter rectification experiment, we can obtain the trend of the retailers’ inventory change before and after the parameter rectification is conducted as shown in Figure 8. The inventory change of the retailer link under the initial parameters of the optimization model is shown in Figure 8a, and it can be found that the system takes a longer time to reach the stabilization condition, and the effect is poor after stabilization, and the inventory fluctuation is large. To make the optimization model achieve a better control effect, we finally determine the optimal combination of parameters after a lot of experimental adjustments, as shown in Table 2.

After the parameters are rectified, we can obtain the inventory change of the retailer link as shown in Figure 8b. Through the comparative analysis, we can find that the time for the system to reach stability is shortened, and the problem of large inventory fluctuation that occurs after the supply chain system is stabilized is substantially reduced.

**Figure 9:**
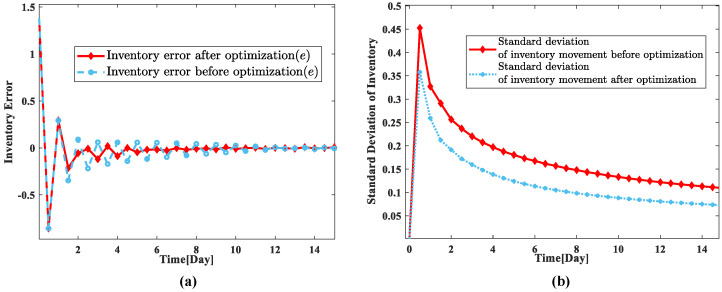
Comparison of inventory error and inventory movement standard deviation before and after parameter tuning. (**a**) Inventory error; (**b**) inventory movement standard deviation.

To reflect more intuitively the control effect of the optimized model before and after the parameter tuning experiment, this paper analyzes the inventory error and the standard deviation of inventory movement, and the results are shown in Figure 9. From Figure 9, it can be found that the inventory error is significantly reduced and the inventory shift standard deviation is also relatively reduced after the parameter tuning optimization, which indicates that the control effect of the optimized model is better after the parameter tuning optimization.

In summary, the results of the control performance parameters of the optimized model before and after parameter tuning can be obtained as shown in Table 3.

### 5.2. Simulation Comparison Analysis

**Figure 10:**
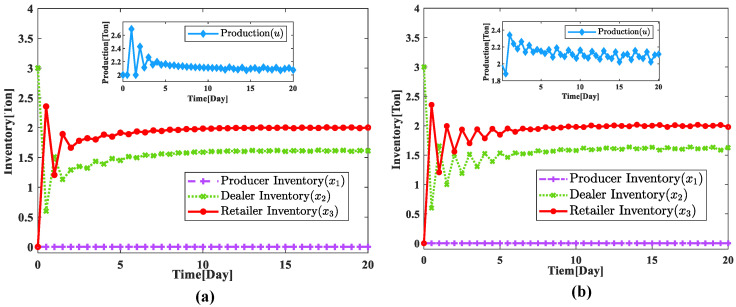
Comparison of supply chain system inventory for different simulation conditions under normal conditions. (**a**) Improved adaptive sliding mode control; (**b**) traditional sliding mode control.

Based on the production change optimization model of the supply chain system under unexpected emergencies after parameter rectification for numerical simulation comparison experiments, this paper mainly conducted two kinds of simulation comparison experiments for the supply chain system, which are: the simulation of the supply chain system under normal conditions and the simulation of the supply chain system under unexpected emergencies. At the same time, to reflect the superiority of the improved sliding mode control, the simulation of the supply chain system for the normal case mainly includes the normal case using the improved adaptive sliding mode control and the control using the traditional sliding mode control. The model parameters of the three-stage supply chain inventory system are the same as those of the parameter rectification optimization experiment, only the market demand it faces has been changed from d=1.5 to d=2. According to the above experimental conditions, we can obtain the inventory changes of each link of the supply chain system under different simulation conditions under normal conditions by experimental simulation as shown in Figure 10.

By analyzing Figure 10, we can find that the improved adaptive sliding mode control has better control effects both in terms of control time and inventory fluctuation after stabilization, and the improved adaptive sliding mode control can shorten the time for the supply chain system to reach the stable state, and at the same time can reduce the problem of inventory fluctuation after system stabilization and reduce the waste of inventory resources of the enterprise. The experimental results show that the improved adaptive sliding mode control is better than the traditional sliding mode control in inventory management and production planning of the supply chain system.

**Figure 11:**
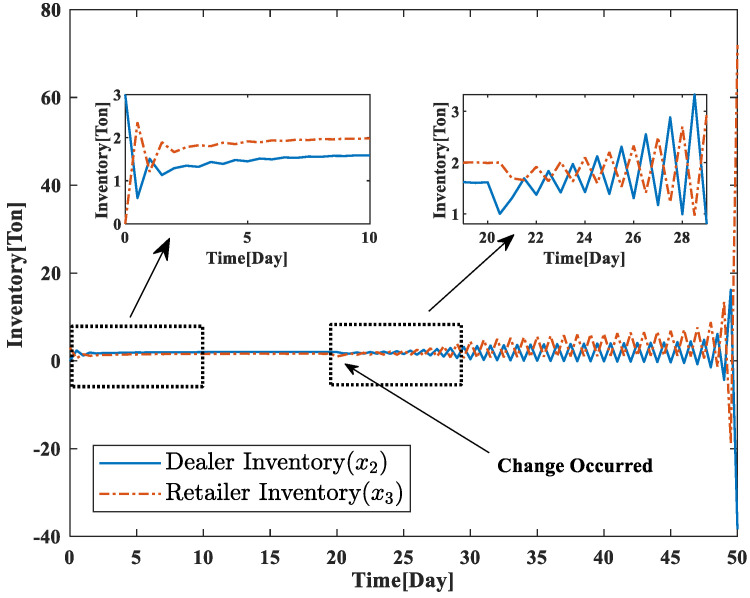
Supply chain system inventory status without optimization model .

To analyze the supply chain system under the contingency situation, this paper considers the change of the distributor link caused by the contingency event, which leads to the change of the relevant parameters of the nonlinear supply chain system. We assume that the change in the distributor chain occurs at the time of t=20, the inventory of goods in the new distributor chain is ${\hat{x}}_{2}=1$, and the parameter of the nonlinear supply chain system with the downstream retailers is $\hat{f}$. If the production volume *u* of the manufacturer does not change accordingly, it can be seen from Figure 11 that the inventory levels of the distributor and retailer segments of the supply chain system do not converge, which indicates that the entire supply chain system segment is unstable and cannot meet the market demand properly at this time.

**Figure 12:**
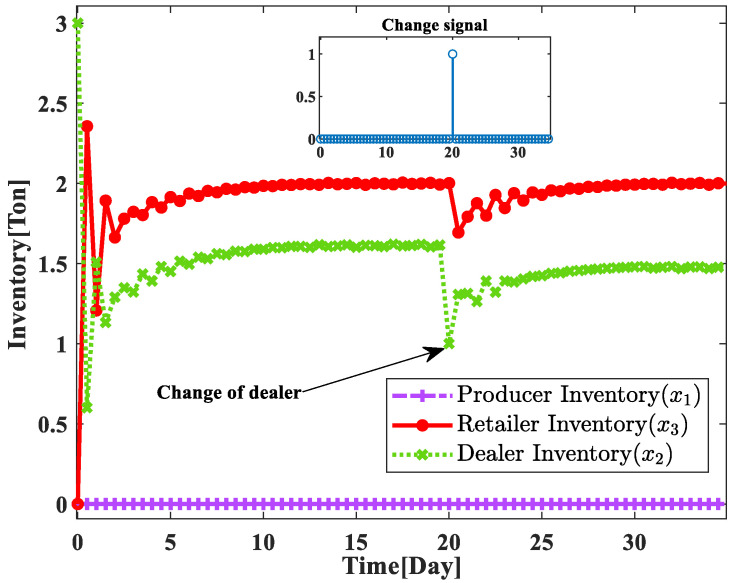
Supply chain system inventory status with optimization model.

After the nonlinear supply chain system optimization model under unexpected emergencies constructed in this paper is able to use RBF neural networks to approximate the changed nonlinear parameters, and then using the improved adaptive sliding mode control to obtain the changed production quantity $\hat{u}$, it can be obtained that the designed controller can still autonomously help the system to make a reasonable production plan after the change of the supply chain system link, and help the system to smoothly carry out the link change, and still keep the supply chain system stable under the unexpected event. In this case, the change in the inventory status of the supply chain system is shown in Figure 12.

By analyzing Figure 12, it can be obtained that the changed supply chain system can be restored to a stable state within 5 days, which indicates that the optimization model constructed in this paper can use the improved adaptive sliding mode control based on RBF neural network to design the production plan of the changed supply chain system, which can autonomously train the nonlinear parameters of the changed supply chain system in a short time, and then help the manufacturer to make a reasonable production plan to help the supply chain system to quickly resume the product supply and restore the supply chain system to a stable state.

## 6. Conclusions

To improve the robustness of the supply chain system when “link change” occurs under unexpected emergencies, this paper conducts in-depth research and simulation analysis on the three-level nonlinear single-chain supply chain production change problem under the action of unexpected emergencies. Compared to traditional sliding mode control, it performs better in supply chain system management optimization, reducing 37.50% of reconciliation time and 42.97% of inventory fluctuation, respectively; the production change optimization model of a nonlinear supply chain system under unexpected emergencies designed in this paper can help the supply chain system to quickly respond to the “link change” brought by unexpected emergencies, help the enterprise to formulate a more reasonable production plan in 5 days, reduce the inventory backlog, and reduce the change risk and resource waste of the enterprise. This can reduce the risk of change and waste of resources, and improve the risk response capability of the supply chain system. The method proposed in this paper aims to help enterprise managers to propose production solutions for “link change” in unexpected situations, and to provide a theoretical basis for optimal management of the supply chain system after “link change” occurs. Of course, the main object of this paper is a three-level single-chain nonlinear supply chain system, while there are many mesh multi-level supply chain systems in real life, whose nonlinearity will also be relatively more complex, which is our next key research direction.

## Figures and Tables

**Table 1:** Initial parameters of the optimization model.

Parameters	*c*	$k_{1}$	$k_{2}$	α	*a*
Set	2.5	0.8	0.3	0.1	20

**Table 2:** Optimal parameters of the optimization model.

Parameters	*c*	$k_{1}$	$k_{2}$	α	*a*
Set	2.0	0.5	0.1	0.05	50

**Table 3:** Optimization model related experimental parameters comparison.

Parameters	Adjustment Time [Day]	MIMSD [T]	MSFS [T]	MIES [T]
Initial Parameters	5	0.4524	0.1061	0.0429
Optimal parameters	8	0.2580	0.0467	0.0184

MISD: Maximum inventory movement standard deviation; MSFS: Maximum stock fluctuation after stabilization; MIES: Maximum inventory error after stabilization.

## Data Availability

This article does not use public data. Only a few examples of assumptions were used and no data need to be shared.
